# Deconstructing Complex Multimorbidity in the Very Old: Findings from the Newcastle 85+ Study

**DOI:** 10.1155/2016/8745670

**Published:** 2016-01-13

**Authors:** Joanna Collerton, Carol Jagger, Mohammad E. Yadegarfar, Karen Davies, Stuart G. Parker, Louise Robinson, Thomas B. L. Kirkwood

**Affiliations:** ^1^Newcastle University Institute for Ageing, Campus for Ageing and Vitality, Newcastle upon Tyne NE4 5PL, UK; ^2^Institute of Health and Society, Newcastle University, Baddiley Clark Building, Richardson Road, Newcastle upon Tyne NE2 4AX, UK; ^3^Institute for Cell and Molecular Bioscience, Newcastle University, Framlington Place, Newcastle upon Tyne NE2 4HH, UK

## Abstract

*Objectives*. To examine the extent and complexity of the morbidity burden in 85-year-olds; identify patterns within multimorbidity; and explore associations with medication and healthcare use.* Participants*. 710 men and women; mean (SD) age 85.5 (0.4) years.* Methods*. Data on 20 chronic conditions (diseases and geriatric conditions) ascertained from general practice records and participant assessment. Cluster analysis within the multimorbid sample identified subgroups sharing morbidity profiles. Clusters were compared on medication and healthcare use.* Results*. 92.7% (658/710) of participants had multimorbidity; median number of conditions: 4 (IQR 3–6). Cluster analysis (multimorbid sample) identified five subgroups sharing similar morbidity profiles; 60.0% (395/658) of participants belonged to one of two high morbidity clusters, with only 4.9% (32/658) in the healthiest cluster. Healthcare use was high, with polypharmacy (≥5 medications) in 69.8% (459/658). Between-cluster differences were found in medication count (*p* = 0.0001); hospital admissions (*p* = 0.022); and general practitioner (*p* = 0.034) and practice nurse consultations (*p* = 0.011). Morbidity load was related to medication burden and use of some, but not all, healthcare services.* Conclusions*. The majority of 85-year-olds had extensive and complex morbidity. Elaborating participant clusters sharing similar morbidity profiles will help inform future healthcare provision and the identification of common underlying biological mechanisms.

## 1. Introduction

The concept of multimorbidity, the cooccurrence of two or more chronic diseases in an individual [1], is attracting increasing research and clinical interest (the related term “comorbidity” is reserved for morbidity cooccurring in relation to a specific index disease [2]). Prevalence estimates for multimorbidity range from 20 to 30% in “all age” populations and are as high as 55–98% in older populations [3]. The cooccurrence of multiple diseases is associated with numerous adverse outcomes including disability, poor quality of life, high healthcare use, and mortality [3, 4]. The provision of effective and cost-effective care for people with multimorbidity presents a major challenge for healthcare systems worldwide and is the subject of on-going debate [5–8]. In the setting of multiple diseases, current approaches to chronic disease management—based largely on the single disease paradigm—can result in complex, fragmented, costly, and potentially ineffective (or even injurious) care [9, 10].

Most multimorbidity research to date has focused on measures based on a simple disease count [11], and there is limited data on how and why particular conditions cooccur and the specific combinations or patterns found. Improved understanding of such patterns would inform the development of better healthcare for patients with multimorbidity and facilitate the identification of common underlying biological mechanisms thereby potentially leading to novel preventive and therapeutic measures [12].

People aged 85 years and over comprise the most rapidly expanding age group in most parts of the world [13]. Whilst multimorbidity is the norm in the very old [14, 15], there is little detailed information on the morbidity profiles found in this age group. In this paper we examine the extent and complexity of the morbidity burden in a population-based sample of 85-year-olds (using the concepts of comorbidity and multimorbidity), identify patterns of morbidity, and explore associations between morbidity profiles and medication and healthcare use. To study morbidity within a population requires comprehensive data on a representative group, which in the case of very old people is rarely available given the inherent difficulty in working with this potentially frail and vulnerable group. We used data from the Newcastle 85+ Study, a population-based cohort study capturing detailed information on the health of a large, representative sample who were all aged 85 at baseline [16, 17]. Considerable effort was invested to secure inclusion of the notably hard-to-reach groups, particularly those living in care homes or with dementia [18]. A novelty of our approach is the use of cluster analysis to identify distinct subgroups of participants with similar combinations of conditions. Furthermore, we included not only chronic diseases but also geriatric syndromes and impairments. Such conditions are as prevalent as chronic diseases in older people and have a marked effect on quality of life, disability, institutionalisation, healthcare use [19], and quality of care [20]. However, they fall outside the disease-focused medical model and have seldom been included in multimorbidity measures.

## 2. Materials and Methods

### 2.1. Study Population

Full details of the Newcastle 85+ Study have been reported [16–18]. In brief, members of the 1921 birth cohort living in Newcastle upon Tyne or North Tyneside (North East England) were recruited at around age 85 using general practice patient lists as the sampling frame. People living in institutions and those with cognitive impairment were included. Recruitment and baseline assessment took place over a 17-month period in 2006-2007.

### 2.2. Study Protocol

Comprehensive measures of health were collected at baseline across multiple clinical, biological, and psychosocial domains. A health assessment—comprising questionnaires, measurements, function tests, and a fasting blood sample—was carried out in the participant's usual residence by a research nurse. General practice medical records were reviewed for diagnosed diseases, prescribed medication, and use of general practice services. In the UK, patients are registered with a single general practice which acts as a gatekeeper to secondary care and receives details of all hospital admissions and outpatient attendances. The review of general practice records included hospital correspondence to ensure that all recorded disease diagnoses were extracted, irrespective of where and when the diagnosis was made.

### 2.3. Diseases and Geriatric Syndromes/Impairments Examined

Fifteen chronic diseases and five geriatric syndromes or impairments (hereafter termed “geriatric conditions”) were selected for investigation. The selection criteria included known impact on morbidity, mortality, and/or healthcare use; availability in the baseline Newcastle 85+ Study dataset; prevalence greater than 3% at study baseline; and less than 10% missing values.  Table 1 lists the 20 conditions examined, together with data sources and ascertainment criteria [21–25]. A systematic review by Diederichs et al. [26] recommended the inclusion of 11 core conditions in any multimorbidity measure, of which we included 10. We were unable to include depression due to the high proportion (15%) of participants with missing data for the depression measure used (15 item Geriatric Depression Scale, GDS-15 [27]); this was mainly because the GDS cannot be used in people with severe cognitive impairment. We included the majority of the chronic conditions prioritised by the UK NHS Quality and Outcomes Framework for General Practice [28].

### 2.4. Medication

Data on prescribed medication was extracted from the general practice records; all participant medication prescribed for use in the month prior to the health assessment was recorded. A count of medications was created after first excluding items such as seasonal vaccinations, diagnostic/monitoring agents, wound-management products, and catheter/stoma products.

### 2.5. Use of Healthcare Services

Data on all consultations with general practitioners and general practice employed nurses (other community nurses were not included) was obtained from the general practice records; a timeframe of 12 months prior to the health assessment was used. Only contacts with the participant's registered general practice were recorded; contacts with externally provided “out of hours” general practice services were excluded. Data on overnight hospital admissions and contacts with outpatient and “Accident and Emergency” services and “Day Hospital” and other intermediate care services was obtained by questionnaire (administered by the research nurse as part of the health assessment). A timeframe of three months was used for outpatient and “Accident and Emergency” services and 12 months for overnight hospital admissions and intermediate care services.

### 2.6. Other Measures

Data on disability level was obtained by nurse-administered questionnaire. A disability score (maximum 17) was calculated from the total number of activities of daily living performed with difficulty or requiring an aid/appliance or personal help [17].

### 2.7. Ethical Approval

The research complied with the requirements of the Declaration of Helsinki. Ethical approval was obtained from the Newcastle and North Tyneside 1 Research Ethics Committee (reference number 06/Q0905/2). Written informed consent was obtained from participants; where people lacked capacity to consent, for example, because of cognitive impairment, a formal written opinion was sought from a relative or carer as previously reported [18].

### 2.8. Statistical Analysis

We first compared the sample with complete data on all 20 conditions (analytic sample) to the sample without complete data. Mann-Whitney *U* tests were used for nonnormally distributed continuous variables and ordinal variables (disability score, education), and *χ* ^2^ tests for categorical variables (sex, place of residence, and prevalence of individual conditions). Sex differences in the prevalence of individual conditions and in multimorbidity were examined by *χ* ^2^ tests and sex differences in the total number of conditions by Mann-Whitney *U* tests. Cluster analysis was used in the sample with multimorbidity (*N* = 658) to identify distinct subgroups of participants with similar combinations of conditions. We first computed a dissimilarity matrix, based on Jaccard's similarity coefficient, on participants' morbidity profiles, and then performed an agglomerative hierarchical cluster analysis [29] using the Calinski/Harabasz index to identify the optimal number of clusters. To characterise between-cluster differences in morbidity profiles, we compared the prevalence of each condition within a specific cluster to that in the total sample with multimorbidity. We defined “higher than average prevalence” as a ratio of prevalence in the cluster to prevalence in the total sample of 1.2  : 1 or higher and “lower than average prevalence” as a ratio of 0.8  : 1 or lower. Clusters were compared by *χ* ^2^ tests for sex distribution, place of residence, and healthcare variables (any use) and by Kruskal-Wallis tests for number of medications and healthcare variables (number of contacts/length of hospital stay). Analyses were performed using STATA version 12.0.

## 3. Results

### 3.1. Sample Selection

Details of sample selection for the Newcastle 85+ Study have been reported [17] (and see the Appendix; see Supplementary Material available online at http://dx.doi.org/10.1155/2016/8745670). The recruited cohort was sociodemographically representative of the local population and of England and Wales, including the proportion in care homes [17]. The present analysis required data from both the health assessment and review of general practice records which was available for 845 participants, 58.2% (845/1453) of those eligible to participate. Complete data on all 20 conditions was available for 710 of these participants (84.0%) who formed the sample for the principal analyses (Appendix, Supplementary Figure 1). Missing data arose from noncompletion of questionnaires, electrocardiograms or blood tests. Comparison of the groups with and without complete data showed that those with missing data were more likely to be female, to be resident in an institution, to have a higher prevalence of osteoporosis, urinary incontinence, and cognitive impairment, and to be more disabled than those with complete data (Appendix, Supplementary Table 1).

### 3.2. Sample Characteristics

Of the 710 participants with complete data on all 20 conditions, the mean (standard deviation) age was 85.5 (0.4) years, 59.9% (425/710) were women and 99.6% (707/710) were of white ethnicity, reflecting the norm for a UK population of this age (Table 2). The majority (80.7%, 573/710) were living in standard (nonsupported) housing, with 13.4% (95/710) in sheltered accommodation and 5.9% (42/710) in an institution (all care homes). Of those not living in an institution, 60.6% (404/667) were living alone.

### 3.3. Prevalence of 20 Diseases and Geriatric Conditions

Hypertension (57.8%, 410/710), osteoarthritis (57.0%, 405/710), and ischaemic heart disease (36.1%, 256/710) were the most prevalent diseases. Hearing impairment (60.4%, 429/710), visual impairment (36.2%, 257/710), and urinary incontinence (31.3%, 222/710) were the most prevalent geriatric conditions (Table 2). Women had a significantly higher prevalence of osteoarthritis, osteoporosis, thyroid disease, and urinary incontinence than men, whilst men had a higher prevalence of atrial fibrillation/flutter and hearing impairment (Appendix, Supplementary Table 2).

### 3.4. Comorbidity for Each of the 20 Diseases and Geriatric Conditions

**Figure 1:**
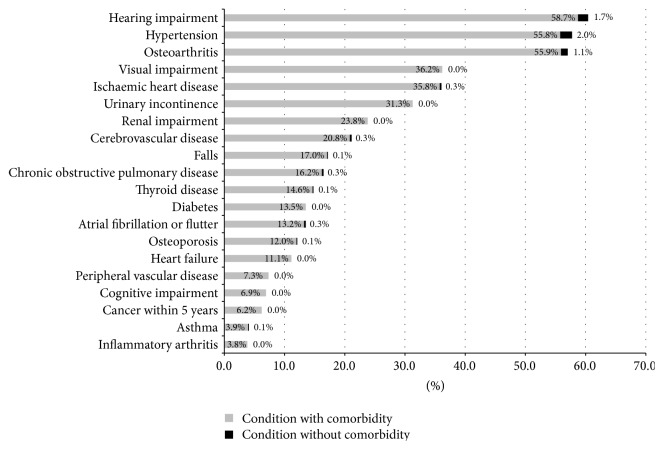
Prevalence of 20 diseases and geriatric conditions, with comorbidity (grey) and without comorbidity (black), in complete case sample (*N* = 710).

Figure 1 shows the prevalence of each of the 20 conditions with and without comorbidity, that is, the cooccurrence of at least one other condition. Supplementary Table 3 (Appendix) shows, for each index condition, the proportion of cases with comorbidity together with the median number of cooccurring conditions (for cases with at least one cooccurring condition). We present the data both including and excluding geriatric conditions in the definition of cooccurring condition. Individual diseases and geriatric conditions very rarely occurred in isolation. When geriatric conditions were included as cooccurring conditions, over 96% of cases of any index condition had at least one other cooccurring condition. The median (interquartile range, IQR) number of cooccurring conditions ranged from 4 (3–5) for hypertension, osteoarthritis, visual impairment, and hearing impairment up to 6 (4–7) for heart failure. Excluding geriatric conditions from the definition of cooccurring condition generally had little effect on the proportion of disease cases with comorbidity; the median (IQR) number of cooccurring diseases ranged from 2 (2–4) for hypertension to 4 (3–5) for heart failure and cancer (within five years).

### 3.5. Total Count of Diseases and Geriatric Conditions

The median total number of conditions (diseases and geriatric conditions) per participant was 4 (IQR, 3–6) and this was higher in women (median 5, IQR 3–6) than men (median 4, IQR 3–6); *p* value = 0.01. The median number of diseases was 3 (IQR 2–4) and for geriatric conditions it was 1 (IQR 1-2). Less than 1% (6/710) of participants had none of the 20 conditions and 6.5% (46/710) had only one condition, whilst 8.9% (63/710) had 8 or more conditions. The prevalence of multimorbidity (two or more conditions) was 92.7% (658/710) and was slightly, but not significantly, higher in women (93.6%, 398/425) than men (91.2%, 260/285); *p* value = 0.225.

### 3.6. Clusters of Participants with Similar Morbidity Profiles

**Figure 2:**
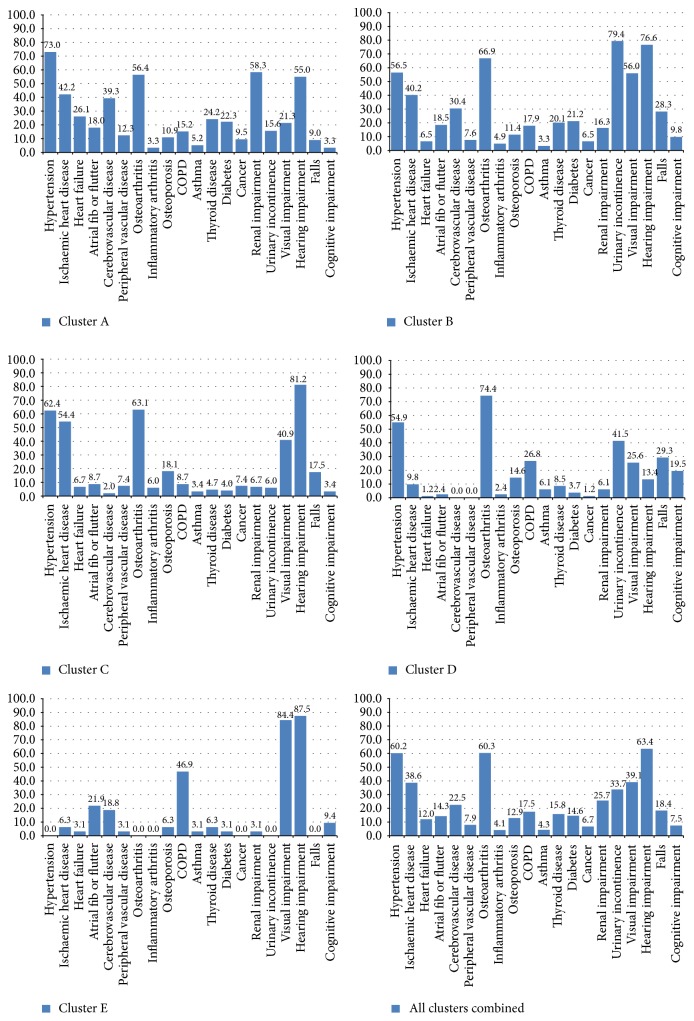
Prevalence of 20 diseases and geriatric conditions in each cluster group, and in total sample with multimorbidity.

The *F*-statistic implied that the optimal number of clusters lays between four and six, and subjective review suggested that a five-cluster solution would yield groups of most clinical relevance. The five clusters varied in prevalence within the multimorbid sample; sex distribution; morbidity profile and the mix found between diseases and geriatric conditions; and use of healthcare services and prescribed medication.  Table 3 provides summary details of the cluster groups, ordered and labelled alphabetically by cluster prevalence.  Table 4 lists condition prevalence by cluster, highlighting those conditions occurring at higher and lower than average prevalence (bold text = higher, ratio of prevalence in cluster to prevalence in total sample with multimorbidity ≥1.2  : 1; italic text = lower, ratio ≤0.8  : 1).  Figure 2 shows the prevalence of the 20 conditions within each of the five clusters and in the total sample with multimorbidity.

The most common clusters—A (32.1% of multimorbid sample, 211/658) and B (28.0%, 184/658)—were both characterised by very high morbidity (10 conditions occurring at higher than average prevalence). The pattern in Cluster A was disease-based, whilst Cluster B had a mix of diseases and geriatric conditions. In Cluster A, 10 diseases (hypertension, heart failure, atrial fibrillation/flutter, cerebrovascular disease, peripheral vascular disease, renal impairment, diabetes, asthma, thyroid disease, and cancer) occurred at higher than average prevalence, whilst most of the geriatric conditions occurred at lower than average prevalence. In contrast, in Cluster B five diseases occurred at higher than average prevalence (atrial fibrillation/flutter, cerebrovascular disease, diabetes, inflammatory arthritis, and thyroid disease), together with all five geriatric conditions. Clusters C (22.6% of sample, 149/658) and D (12.5%, 82/658) were characterised by intermediate morbidity; four and six conditions, respectively, occurred at higher than average prevalence, comprising a mix of diseases and geriatric conditions. Cluster E (4.9% of sample, 32/658), the least common group, appeared to be the healthiest cluster; whilst five conditions occurred at higher than average prevalence (mix of diseases and geriatric conditions), 14 of the 20 conditions occurred at zero or low prevalence. Higher than average prevalence was found for three diseases (ischaemic heart disease, inflammatory arthritis, and osteoporosis) and one geriatric condition (hearing impairment) in Cluster C; three diseases (osteoarthritis, chronic obstructive pulmonary disease, and asthma) and three geriatric conditions (urinary incontinence, falls, and cognitive impairment) in Cluster D; and two diseases (atrial fibrillation/flutter and chronic obstructive airways disease) and three geriatric conditions (visual impairment, hearing impairment, and cognitive impairment) in Cluster E. Four conditions—hypertension, osteoarthritis, hearing impairment, and visual impairment—occurred at high prevalence in at least four of the five clusters.

The total number of conditions amongst cluster group members reflected the cluster morbidity profile; Clusters A and B had the highest total number of conditions (medians of five and six, resp.) with Clusters C, D, and E having lower numbers (medians of four, three, and three, resp.). There was a significant difference in sex distribution between the clusters (*p* value = 0.002). Overall, women comprised 60.5% (398/658) of the total sample with multimorbidity, whereas Cluster E had equal numbers of men and women and in Cluster D the proportion of women was 75.6% (62/82). Only 6.4% (42/658) of participants with multimorbidity were living in an institution (all in care homes); the prevalence was somewhat higher in Clusters B (9.8%, 18/184) and D (9.8% 8/82), *p* value = 0.056, which may reflect the high proportion with cognitive impairment in those clusters.

### 3.7. Medication and Healthcare Use

Participants with multimorbidity were high consumers of healthcare, particularly primary care (Table 5). Prescribed medication burden was also high, with polypharmacy (five or more medications) in 69.8% (459/658) of participants and 17.3% (114/658) prescribed 10 or more medications. Between-cluster differences were found in the number of medications (*p* value = 0.0001); overnight hospital admissions (proportion admitted at least once in previous 12 months, *p* value = 0.022); general practitioner consultations (proportion consulting at least once in previous 12 months, *p*-value = 0.034); and general practice nurse consultations (proportion consulting at least once in previous 12 months, *p* value = 0.011 plus number of consultations for those consulting, *p* value = 0.009). For medication, hospital admissions, and general practice nurse consultations, the level of use generally reflected cluster morbidity load with higher use found in Clusters A and B. In those with at least one hospital admission, there was some suggestion of a higher total length of stay in Cluster B, although the difference did not reach statistical significance (*p* value = 0.058). Whilst there were cluster differences in the proportion consulting their general practitioner at least once during the previous 12 months, the high percentage found in all clusters (87.8–97.3%) makes it difficult to determine whether this variation is of clinical significance. The number of general practitioner contacts, amongst those who consulted, was similar across clusters. Given the difference in sex distribution between clusters and that we have previously found sex differences in general practice nurse consultations in this cohort (women having lower levels of use than men) [17], we repeated the analysis of general practice nurse consultations adjusting for sex. Between-cluster differences remained in both the proportion consulting (*p* value = 0.026) and the number of consultations (*p* value < 0.001).

## 4. Discussion

We have reported novel data detailing the extensive and complex morbidity burden found in a UK population-based cohort of 85-year-olds and the relationship between morbidity profiles and medication and healthcare use. Novel aspects of our approach include the use of cluster analysis to identify distinct subgroups of participants with similar combinations of conditions and the inclusion of geriatric syndromes and impairments in addition to diseases. We found that chronic diseases and geriatric conditions were both common in the very old and that individual conditions very rarely occurred in isolation. Multimorbidity was almost universal and the average number of conditions was high. Cluster analysis identified five distinct subgroups of participants with similar patterns of morbidity. The two most prevalent clusters, accounting for 60% of the sample, showed very high levels of morbidity; one was predominantly disease-based, whilst the other comprised a mix of diseases and geriatric conditions. The healthiest profile accounted for only 5% of the sample and, even in this “healthy” cluster, participants still had an average of three conditions. Participants with multimorbidity were high consumers of healthcare, particularly primary care, and prescribed medication burden was high with polypharmacy (five or more prescribed medications) found in almost 70%.

It should be noted that cluster analysis is an exploratory technique and different clustering algorithms can produce varying results [30]. However, our findings of disease combinations which mirror known groupings and the between-cluster differences in healthcare use provide evidence of the validity of our approach. Cluster A included five interlinked “circulatory” diseases (hypertension, heart failure, atrial fibrillation/flutter, cerebrovascular disease, and peripheral vascular disease) and three diseases associated with circulatory disease (diabetes, thyroid disease, and renal impairment). Cluster A also included cancer which could be linked to circulatory diseases through the common risk factor of smoking. Cluster B included the established groupings of atrial fibrillation/flutter with cerebrovascular disease and with thyroid disease and diabetes with cerebrovascular disease. Another recognised pairing was that of atrial fibrillation and cognitive impairment found in Clusters B and E. Geriatric syndromes tended to cluster together in line with previous reports [31, 32]. All five geriatric conditions occurred at higher than average prevalence in Cluster B and three conditions in Clusters D and E; in contrast, most geriatric conditions were less prevalent in Cluster A. Geriatric syndromes are thought to result from impairments across multiple systems; they may share common risk factors and pathophysiological mechanisms and could be amenable to unified intervention strategies [19]. Of note, Clusters C, D, and E included less familiar disease groupings, for example, ischaemic heart disease, inflammatory arthritis, and osteoporosis (Cluster C), and some diseases in Cluster A (asthma) and Cluster B (inflammatory arthritis) do not readily “fit” with the rest of the cluster. Disentangling the basis of such “unfamiliar” associations may point the way to promising new avenues of research [33].

Recent systematic reviews of studies of multimorbidity patterns confirm the paucity of research in this area, particularly in the very old [33, 34]. Cluster analysis has been used in a small number of studies [30, 35–41], many of which focused on specific groups such as the hospitalised elderly [37], Native Americans [38], US Veterans [39], and homeless veterans [40]. Most studies used the approach of clustering by condition rather than by participant [35–39, 41]; this produces somewhat crude groupings and has the drawback that each condition can only appear in one cluster (an artefact of the clustering algorithm [39]), as well as it being less straightforward to assign study participants to cluster groups and therefore to examine associations with outcomes. Only two studies have, as we did, clustered by participant to identify distinct subgroups of people sharing similar morbidity profiles, neither of which used population-based samples [30, 40].

Few studies have focused on the very old, all of which used cluster analysis with clustering by condition. Marengoni et al. examined morbidity patterns in the Kungsholmen study (*n* = 1077, aged 77 and over) [36]; Formiga et al. in the Octobaix study (*n* = 328 aged 85) [41]; and Dong et al. in the ELSA 85 study (*n* = 496, aged 85) [35]. Five clusters were identified in the Kungsholmen cohort: circulatory; cardiopulmonary; dementia, depression and hip fracture; diabetes and visual impairment; and cancer with anaemia [36]. The Octobaix cohort had four main clusters: circulatory plus visual impairment; dementia, Parkinson's disease, peripheral vascular disease, dyslipidaemia, and anaemia; chronic obstructive pulmonary disease and malignancy; and hearing impairment [41]. Five main clusters were found in women from the Elsa 85 cohort: vascular; cardiopulmonary; dementia and affective disorders; osteoarthritis and urinary incontinence; and malignancy and thyroid disease [35]. Our study builds on these findings by including a larger sample size of the very old and a larger number of conditions (with osteoarthritis, incontinence and falls included) and by using an alternative approach of clustering by participant rather than by condition. Marked methodological heterogeneity between studies makes direct comparison of the patterns problematic; however the finding of circulatory cluster(s) is a common theme across all studies of the very old, including our own.

Studies of multimorbidity patterns, in all age groups, were the focus of a recent systematic review by Prados-Torres et al. [33]. Fourteen studies were included, 8 of which focused on participants over 60 years whilst 3 included individuals as young as 15; the Kungsholmen study was the only study focusing on the very old [36]. Ninety-seven disease patterns were identified across the 14 studies. The considerable methodological variation between studies—in age group, setting, number and types of conditions included, ascertainment criteria, and statistical techniques—makes direct comparison difficult. Nevertheless, three broad groups of patterns were highlighted: a cardiovascular/cardiometabolic group, found in 10/14 studies; a mental health group, in 10/14 studies (at least one mental health problem, most commonly depression and/or anxiety); and a musculoskeletal group, in 10/14 studies (at least one musculoskeletal condition, most commonly arthropathy, back/neck pain, and/or osteoporosis). In each of these broad groups, a wide range of additional comorbidities was found, only some of which had logical associations. Comparing these findings to studies of the very old, all three broad groups can be seen in the Elsa 85 cohort [35], two in the Kungsholmen cohort [36], and one in the Octobaix cohort [41]. In the Newcastle 85+ cohort, our finding of a cardiometabolic cluster (Cluster A), together with musculoskeletal conditions in Clusters C and D, would fit with these broad trends, although we found osteoarthritis to be of high prevalence in four of our five clusters. We were unable to include measures of mental health in our analysis. Whilst it would be interesting to further analyse pattern differences between the very old and younger age groups, the marked methodological differences between studies precludes meaningful interpretation.

Strengths of this study include its population-based sample, which included the institutionalised and those with cognitive impairment, and the domiciliary assessment which avoids the selection bias inherent in clinic-based assessment of this age group. The use of dual data sources is a further strength; disease ascertainment from medical records is more reliable than self-report in older age groups, particularly in those challenged by multimorbidity or cognitive impairment [42–44], whilst participant assessment is superior for geriatric conditions which may be undiagnosed and/or their presence poorly documented [45]. Our work has a number of limitations. The sample analysed (*n* = 710) represents 49% of those eligible to participate. Within the limits of the analysis possible, it does not appear that study nonparticipants were less healthy than participants although those with cognitive impairment may have been underrepresented [46]. However, those participants excluded from the analysis due to missing data were less healthy than those with complete data, and consequently our data may underestimate the scale of multimorbidity. Some important conditions were excluded due to absence in the study dataset or a high rate of missing values, for example, mental health problems; hence our estimate of multimorbidity is somewhat conservative. Our sample derives from a single urban area in North East England, with predominantly white ethnicity. Whilst 85-year-olds in this area are sociodemographically and ethnically similar to those in England and Wales as a whole [17], they may differ from those in other parts of the world.

The extensive and complex morbidity burden found in the majority of very old people presents a considerable challenge for healthcare services. Current approaches to chronic disease management are focused largely on a single disease paradigm. In patients with many conditions, application of multiple disease-specific guidelines can lead to clinical chaos, polypharmacy, and interactions between strategies for individual conditions [47, 48]. Healthcare can become fragmented, costly, and potentially ineffective (or even injurious) [9, 10]. Despite growing recognition of the importance of multimorbidity, there remains insufficient data to inform evidence-based care for multimorbid patients of any age [49] and the knowledge gap is particularly acute in older people [3]. Clinical trials routinely exclude patients with cooccurring conditions [50], and older people are consistently underrepresented [50, 51]. Clinical practice guidelines focused on the index disease fail to address the needs of people with complex multimorbidity [47, 48, 52]; furthermore they rarely include information on the quality of research evidence in older people or give specific recommendations for older people [48, 53]. Strategies proposed to improve the care of patients with multimorbidity [5–7, 54–58] will need to be appropriate to the very old who, as we have shown, have a considerable and complex morbidity burden. In the UK, the demarcations between (and within) primary care, community health services, and secondary care and between health and social care are increasingly seen as a barrier to providing the personalised and coordinated approach needed by older people with multimorbidity. The National Health Service is therefore supporting the creation of major new models of care integrated around the patient and their needs, which will cross traditional organisational and departmental boundaries [59].

## 5. Conclusions

The majority of 85-year-olds in this population-based cohort in North East England had extensive and complex morbidity. The elaboration of clusters of older people sharing similar morbidity profiles is likely, in time, to help throw light on shared pathophysiological processes, creating the potential for novel preventive measures and targeted therapies. Furthermore, it will inform the development of healthcare services which are better able to meet the complex needs of the very old.

## Supplementary Material

Supplementary Material contains an appendix to the paper that gives further details of the sample selection and additional results tables (Supplementary Tables 1, 2, and 3).

## Figures and Tables

**Table 1:** 20 diseases and geriatric conditions examined; data sources and ascertainment criteria.

Diseases (15)		
Disease	Data source	Criteria
Hypertension	General practice (GP) records	Documented diagnosis of hypertension regardless of date.
Ischaemic heart disease	GP records and health assessment (HA) electrocardiogram (ECG)	Documented diagnosis of angina or myocardial infarction or coronary artery bypass grafts or coronary angioplasty or coronary stent regardless of date. Participants without a preexisting diagnosis could be additionally assigned on the basis of Minnesota codes [21] commencing 1-1 or 5-1 on 12 lead ECG conducted as part of the health assessment.
Heart failure	GP records	Documented diagnosis of heart failure regardless of date.
Atrial fibrillation or flutter	HA ECG	Minnesota codes 8-3-1 or 8-3-2 on 12 lead ECG conducted as part of the health assessment.
Cerebrovascular disease	GP records	Documented diagnosis of stroke or transient ischaemic attack or carotid endarterectomy regardless of date.
Peripheral vascular disease	GP records	Documented diagnosis of peripheral vascular disease regardless of date.
Osteoarthritis	GP records	Documented diagnosis of osteoarthritis or cervical spondylosis or lumbar spondylosis regardless of date.
Inflammatory arthritis	GP records	Documented diagnosis of rheumatoid arthritis or psoriatic arthropathy or ankylosing spondylitis regardless of date.
Osteoporosis	GP records	Documented diagnosis of osteoporosis regardless of date.
Chronic obstructive pulmonary disease	GP records	Documented diagnosis of chronic obstructive pulmonary disease (COPD) regardless of date.
Asthma	GP records	Documented diagnosis of asthma excluding childhood asthma and excluding asthma in conjunction with COPD.
Thyroid disease	GP records	Documented diagnosis of hypothyroidism or hyperthyroidism regardless of date.
Diabetes mellitus	GP records	Documented diagnosis of diabetes mellitus regardless of date.
Cancer within previous 5 years	GP records	Documented diagnosis of cancer diagnosed within previous 5 years excluding nonmelanoma skin cancer.
Renal impairment	HA serum creatinine	Estimated glomerular filtration rate of less than 45 mL/min/1.73 m^2^ calculated using the Chronic Kidney Disease Epidemiology Collaboration equation [22] using serum creatinine measured as part of the health assessment. This cut point identifies Stages 3B, 4, and 5 Chronic Kidney Disease [23].

Geriatric conditions (5)		
Geriatric condition	Data source	Criteria

Urinary incontinence	HA questionnaire	Moderate, severe, or profound incontinence (classified on basis of frequency of episodes and volume of urine leakage [24]) or catheterised for previous 12 months.
Falls	HA questionnaire	Two or more falls in previous 12 months.
Visual impairment	HA questionnaire	Self-reported difficulty recognizing a friend across the road or reading ordinary newsprint, with aids if worn.
Hearing impairment	HA questionnaire	Self-reported difficulty hearing someone taking in a quiet room or following a conversation with background noise, with aids if worn.
Cognitive impairment	HA cognitive test	Standardised minimental state examination score [25] of 21 or lower.

**Table 2:** Sample characteristics: 710 participants with complete data on all 20 conditions.

Age, mean (SD) years	85.5 (0.4)
Female, % (*n*)	59.9 (425)
White ethnicity, % (*n*)	99.6 (707)
Living arrangements, % (*n*)	
Standard (nonsupported) housing	80.7 (573)
Sheltered housing	13.4 (95)
Institution	5.9 (42)
Years in full-time education, % (*n*)	
0–9	64.7 (458)
10-11	22.7 (161)
12+	12.6 (89)
Diseases, % (*n*)	
Hypertension	57.8 (410)
Ischaemic heart disease	36.1 (256)
Heart failure	11.1 (79)
Atrial fibrillation or flutter	13.5 (96)
Cerebrovascular disease	21.1 (150)
Peripheral vascular disease	7.3 (52)
Osteoarthritis	57.0 (405)
Inflammatory arthritis	3.8 (27)
Osteoporosis	12.1 (86)
Chronic obstructive pulmonary disease	16.5 (117)
Asthma	4.1 (29)
Diabetes mellitus	13.5 (96)
Thyroid disease	14.8 (105)
Cancer within 5 years	6.2 (44)
Renal impairment	23.8 (169)
Geriatric conditions, % (*n*)	
Urinary incontinence	31.3 (222)
Falls	17.2 (122)
Visual impairment	36.2 (257)
Hearing impairment	60.4 (429)
Cognitive impairment	6.9 (49)
Disability score^*∗*^, median (IQR)	3 (1–6)

^*∗*^Total number of activities of daily living performed with difficulty or requiring an aid/appliance or personal help [17].

**Table 3:** Description of five clusters of participants with similar morbidity profiles identified in sample with multimorbidity (*N* = 658).

Cluster	Prevalence within multimorbid sample % (*n*)	Female % (*n*)	Median (IQR) number of conditions	Number of diseases/geriatric conditions occurring at higher than average prevalence^*∗*^	Conditions^†^ occurring at higher than average prevalence: prevalence, % (ratio of prevalence in cluster to prevalence in total multimorbid sample)	Number of diseases/geriatric conditions occurring at lower than average prevalence^*∗*^	Conditions^†^ occurring at lower than average prevalence: prevalence, % (ratio of prevalence in cluster to prevalence in total multimorbid sample)
A	32.1 (211)	56.4 (119)	5 (3–7)	10/0	Hypertension 73.0 (1.2) Renal impairment 58.3 (2.3) Cerebrovascular disease 39.3 (1.8) Heart failure 26.1 (2.2) Thyroid disease 24.2 (1.5) Diabetes mellitus 22.3 (1.5) Atrial fibrillation/flutter 18.0 (1.3) Peripheral vascular disease 12.3 (1.6) Cancer 9.5 (1.4) Asthma 5.2 (1.2)	2/4	*Cognitive impairment 3.3 (0.5)* Inflammatory arthritis 3.3 (0.8) *Falls 9.0 (0.5)* Osteoporosis 10.9 (0.8) *Urinary incontinence 15.6 (0.5)* *Visual impairment 21.3 (0.6)*

B	28.0 (184)	66.3 (122)	6 (5–7)	5/5	*Urinary incontinence 79.4 (2.4)* *Hearing impairment 76.6 (1.2)* *Visual impairment 56.0 (1.4)* Cerebrovascular disease 30.4 (1.4) *Falls 28.3 (1.5)* Diabetes mellitus 21.2 (1.5) Thyroid disease 20.1 (1.3) Atrial fibrillation/flutter 18.5 (1.3) *Cognitive impairment 9.8 (1.3)* Inflammatory arthritis 4.9 (1.2)	3/0	Asthma 3.3 (0.8) Heart failure 6.5 (0.5) Renal impairment 16.3 (0.6)

C	22.6 (149)	53.0 (79)	4 (3–5)	3/1	*Hearing impairment 81.2 (1.3)* Ischaemic heart disease 54.4 (1.4) Osteoporosis 18.1 (1.4) Inflammatory arthritis 6.0 (1.5)	8/2	Cerebrovascular disease 2.0 (0.1) *Cognitive impairment 3.4 (0.5)* Asthma 3.4 (0.8) Diabetes mellitus 4.0 (0.3) Thyroid disease 4.7 (0.3) *Urinary incontinence 6.0 (0.2)* Renal impairment 6.7 (0.3) Heart failure 6.7 (0.6) Chronic obstructive pulmonary disease 8.7 (0.5) Atrial fibrillation/flutter 8.7 (0.6)

D	12.5 (82)	75.6 (62)	3 (2–4)	3/3	Osteoarthritis 74.4 (1.2) *Urinary incontinence 41.5 (1.2)* *Falls 29.3 (1.6)* Chronic obstructive pulmonary disease 26.8 (1.5) *Cognitive impairment 19.5 (2.6)* Asthma 6.1 (1.4)	10/2	Cerebrovascular disease 0.0 (0.0) Peripheral vascular disease 0.0 (0.0) Heart failure 1.2 (0.1) Cancer 1.2 (0.2) Atrial fibrillation/flutter 2.4 (0.2) Inflammatory arthritis 2.4 (0.6) Diabetes mellitus 3.7 (0.3) Renal impairment 6.1 (0.2) Thyroid disease 8.5 (0.5) Ischaemic heart disease 9.8 (0.3) *Hearing impairment 13.4 (0.2)* *Visual impairment 25.6 (0.7)*

E	4.9 (32)	50.0 (16)	3 (2–4)	2/3	*Hearing impairment 87.5 (1.4)* *Visual impairment 84.4 (2.2)* Chronic obstructive pulmonary disease 46.9 (2.7) Atrial fibrillation/flutter 21.9 (1.5) *Cognitive impairment 9.4 (1.3)*	13/2	Hypertension 0.0 (0.0) Osteoarthritis 0.0 (0.0) Inflammatory arthritis 0.0 (0.0) Cancer 0.0 (0.0) *Urinary incontinence 0.0 (0.0)* *Falls 0.0 (0.0)* Renal impairment 3.1 (0.1) Diabetes mellitus 3.1 (0.2) Heart failure 3.1 (0.3) Peripheral vascular disease 3.1 (0.4) Asthma 3.1 (0.7) Ischaemic heart disease 6.3 (0.2) Thyroid disease 6.3 (0.4) Osteoporosis 6.3 (0.5) Cerebrovascular disease 18.8 (0.8)

^*∗*^Higher than average prevalence of a condition defined as a ratio of prevalence in cluster to prevalence in total sample with multimorbidity ≥1.2 : 1. Lower than average prevalence of a condition defined as a ratio of prevalence in cluster to prevalence in total sample with multimorbidity ≤0.8 : 1.

^†^Geriatric conditions (syndromes/impairments) are shown in italic font.

**Table 4:** Prevalence (%) of 20 conditions in each cluster and in total sample with multimorbidity. Conditions occurring at higher than average prevalence^*∗*^ are shown in bold text; those occurring at lower than average prevalence^*∗*^ are shown in italic text.

	Cluster A	Cluster B	Cluster C	Cluster D	Cluster E	Total multimorbid sample
	*n* = 211	*n* = 184	*n* = 149	*n* = 82	*n* = 32	*n* = 658
Diseases						
Hypertension	**73.0**	56.5	62.4	54.9	*0.0*	60.2
Ischaemic heart disease	42.2	40.2	**54.4**	*9.8*	*6.3*	38.6
Heart failure	**26.1**	*6.5*	*6.7*	*1.2*	*3.1*	12.0
Atrial fibrillation or flutter	**18.0**	**18.5**	*8.7*	*2.4*	**21.9**	14.3
Cerebrovascular disease	**39.3**	**30.4**	*2.0*	*0.0*	*18.8*	22.5
Peripheral vascular disease	**12.3**	7.6	7.4	*0.0*	*3.1*	7.9
Osteoarthritis	56.4	66.9	63.1	**74.4**	*0.0*	60.3
Inflammatory arthritis	*3.3*	**4.9**	**6.0**	*2.4*	*0.0*	4.1
Osteoporosis	*10.9*	11.4	**18.1**	14.6	*6.3*	12.9
Chronic obstructive pulmonary disease	15.2	17.9	*8.7*	**26.8**	**46.9**	17.5
Asthma	**5.2**	*3.3*	*3.4*	**6.1**	*3.1*	4.3
Thyroid disease	**24.2**	**20.1**	*4.7*	*8.5*	*6.3*	15.8
Diabetes mellitus	**22.3**	**21.2**	*4.0*	*3.7*	*3.1*	14.6
Cancer within 5 years	**9.5**	6.5	7.4	*1.2*	*0.0*	6.7
Renal impairment	**58.3**	*16.3*	*6.7*	*6.1*	*3.1*	25.7
Geriatric conditions						
Urinary incontinence	*15.6*	**79.4**	*6.0*	**41.5**	*0.0*	33.7
Visual impairment	*21.3*	**56.0**	40.9	*25.6*	**84.4**	39.1
Hearing impairment	55.0	**76.6**	**81.2**	*13.4*	**87.5**	63.4
Falls	*9.0*	**28.3**	17.5	**29.3**	*0.0*	18.4
Cognitive impairment	*3.3*	**9.8**	*3.4*	**19.5**	**9.4**	7.5

^*∗*^Higher than average prevalence of a condition defined as a ratio of prevalence in cluster to prevalence in total sample with multimorbidity ≥1.2 : 1. Lower than average prevalence of a condition defined as a ratio of prevalence in cluster to prevalence in total sample with multimorbidity ≤0.8 : 1.

**Table 5:** Medication and healthcare use in total sample with multimorbidity and cluster groups.

	Total multimorbid sample	Cluster A	Cluster B	Cluster C	Cluster D	Cluster E	*p* value^*∗*^
	(*n* = 658)	(*n* = 211)	(*n* = 184)	(*n* = 149)	(*n* = 82)	(*n* = 32)	
Previous 1 month							
Prescribed medication^†^							
Median (interquartile range) number of items	6 (4–9)	7 (5–9)	7 (4–9)	6 (4–8)	5 (3–7)	3 (1–6.5)	**0.0001**
Previous 3 months							
Any outpatient attendance, % (*n*)^‡^	34.1 (223)	37.1 (78)	35.9 (65)	33.6 (50)	24.4 (20)	31.3 (10)	0.319
Median (interquartile range) number of outpatient attendances^‡,§^	1 (1-2)	1 (1-2)	1 (1-2)	1 (1-2)	1 (1-2)	1 (1-2)	0.859
Any “Accident and Emergency” attendance, % (*n*)^‡^	7.6 (50)	7.1 (15)	7.1 (13)	8.7 (13)	4.9 (4)	15.6 (5)	0.380
Median (interquartile range) number of “Accident and Emergency” attendances^‡,§^	1 (1-1)	1 (1-1)	1 (1-1)	1 (1-1)	1 (1-1)	1 (1-1)	0.609
Previous 12 months							
Any overnight hospital admission, % (*n*)^‡^	22.5 (148)	27.6 (58)	25.0 (46)	20.1 (30)	11.0 (9)	15.6 (5)	**0.022**
Median (interquartile range) total length of stay (days)^‡,§^	7 (3–17)	6 (2–15)	13 (5–23)	5 (2–14)	7 (3–14)	4 (3–7)	0.058
Any “Day Hospital” attendance, % (*n*)^‡^	7.6 (50)	9.1 (19)	7.7 (14)	4.0 (6)	7.3 (6)	16.1 (5)	0.163
Any other intermediate care service contacts, % (*n*)^‡^	7.1 (46)	4.7 (10)	8.9 (16)	10.3 (15)	3.7 (3)	6.5 (2)	0.171
Any consultations with own general practitioner, % (*n*)^†^	93.3 (614)	91.5 (193)	97.3 (179)	93.3 (139)	87.8 (72)	96.9 (31)	**0.034**
Median (interquartile range) number of general practitioner consultations^†,§^	5 (3–9)	6 (3–9)	5 (3–8)	5 (3–9)	5 (3.5–9)	4 (2–9)	0.638
Any consultations with general practice nurse, % (*n*)^†^	81.0 (533)	84.8 (179)	81.5 (150)	83.9 (125)	69.5 (57)	68.8 (22)	**0.011**
Median (interquartile range) number of general practice nurse consultations^†,§^	3 (2–5)	3 (2–6)	2.5 (1–5)	3 (1–5)	2 (1–4)	2 (1–4)	**0.009**

^*∗*^ *p* value for no difference between cluster groups.

^†^Data source: review of general practice records.

^‡^Data source: nurse-administered questionnaire.

^§^Median (interquartile range) reported for those participants with at least one contact with service.

## References

[1] van den Akker M., Buntinx F., Knottnerus J. A. (1996). Comorbidity or multimorbidity: what's in a name? A review of literature. *European Journal of General Practice*.

[2] Feinstein A. R. (1970). The pre-therapeutic classification of co-morbidity in chronic disease. *Journal of Chronic Diseases*.

[3] Marengoni A., Angleman S., Melis R. (2011). Aging with multimorbidity: a systematic review of the literature. *Ageing Research Reviews*.

[4] Boyd C. M., Ritchie C. S., Tipton E. F., Studenski S. A., Wieland D. (2008). From Bedside to Bench: summary from the American Geriatrics Society/National Institute on Aging Research conference on comorbidity and multiple morbidity in older adults. *Aging Clinical and Experimental Research*.

[5] Salisbury C. (2012). Multimorbidity: redesigning health care for people who use it. *The Lancet*.

[6] Mangin D., Heath I., Jamoulle M. (2012). Beyond diagnosis: rising to the multimorbidity challenge. *British Medical Journal*.

[7] American Geriatrics Society Expert Panel on the Care of Older Adults with Multimorbidity (2012). Patient-centered care for older adults with multiple chronic conditions: a stepwise approach from the American Geriatrics Society. *Journal of the American Geriatrics Society*.

[8] Roland M., Paddison C. (2013). Better management of patients with multimorbidity. *British Medical Journal*.

[9] Fortin M., Soubhi H., Hudon C., Bayliss E. A., van den Akker M. (2007). Multimorbidity's many challenges. *British Medical Journal*.

[10] Vogeli C., Shields A. E., Lee T. A. (2007). Multiple chronic conditions: prevalence, health consequences, and implications for quality, care management, and costs. *Journal of General Internal Medicine*.

[11] Huntley A. L., Johnson R., Purdy S., Valderas J. M., Salisbury C. (2012). Measures of multimorbidity and morbidity burden for use in primary care and community settings: a systematic review and guide. *Annals of Family Medicine*.

[12] Marengoni A., Fratiglioni L. (2011). Disease clusters in older adults: rationale and need for investigation. *Journal of the American Geriatrics Society*.

[13] United Nations Department of Economic and Social Affairs. Population Division (2009). *World Population Ageing: 2009*.

[14] Barnett K., Mercer S. W., Norbury M., Watt G., Wyke S., Guthrie B. (2012). Epidemiology of multimorbidity and implications for health care, research, and medical education: a cross-sectional study. *The Lancet*.

[15] Salive M. E. (2013). Multimorbidity in older adults. *Epidemiologic Reviews*.

[16] Collerton J., Barrass K., Bond J. (2007). The Newcastle 85+ study: biological, clinical and psychosocial factors associated with healthy ageing: study protocol. *BMC Geriatrics*.

[17] Collerton J., Davies K., Jagger C. (2009). Health and disease in 85 year olds: baseline findings from the Newcastle 85+ cohort study. *The British Medical Journal*.

[18] Davies K., Collerton J. C., Jagger C. (2010). Engaging the oldest old in research: lessons from the Newcastle 85+ study. *BMC Geriatrics*.

[19] Inouye S. K., Studenski S., Tinetti M. E., Kuchel G. A. (2007). Geriatric syndromes: clinical, research, and policy implications of a core geriatric concept. *Journal of the American Geriatrics Society*.

[20] Min L., Kerr E. A., Blaum C. S., Reuben D., Cigolle C., Wenger N. (2014). Contrasting effects of geriatric versus general medical multimorbidity on quality of ambulatory care. *Journal of the American Geriatrics Society*.

[21] Prineas R. J., Crow R. S., Zhang Z.-M. (2010). *The Minnesota Code Manual of Electrocardiographic Findings*.

[22] Levey A. S., Stevens L. A., Schmid C. H. (2009). A new equation to estimate glomerular filtration rate. *Annals of Internal Medicine*.

[23] Crowe E., Halpin D., Stevens P. (2008). Early identification and management of chronic kidney disease: summary of NICE guidance. *British Medical Journal*.

[24] Mcgrother C. W., Donaldson M. M. K., Shaw C. (2004). Storage symptoms of the bladder: prevalence, incidence and need for services in the UK. *BJU International*.

[25] Molloy D. W., Standish T. I. M. (1997). A guide to the standardized Mini-Mental State Examination. *International Psychogeriatrics*.

[26] Diederichs C., Berger K., Bartels D. B. (2011). The measurement of multiple chronic diseases—a systematic review on existing multimorbidity indices. *Journals of Gerontology Series A: Biological Sciences and Medical Sciences*.

[27] Sheikh J. A., Yesavage J., Brink T. L. (1986). Geriatric Depression Scale (GDS): recent findings and development of a shorter version. *Clinical Gerontology: A Guide to Assessment and Intervention*.

[28] NHS: The Information Centre for Health and Social Care (2014-15). *Quality and Outcomes Framework—Prevalence, Achievements and Exceptions Report*.

[29] Ward J. H. (1963). Hierarchical grouping to optimize an objective function. *Journal of the American Statistical Association*.

[30] Newcomer S. R., Steiner J. F., Bayliss E. A. (2011). Identifying subgroups of complex patients with cluster analysis. *The American Journal of Managed Care*.

[31] Cigolle C. T., Langa K. M., Kabeto M. U., Tian Z., Blaum C. S. (2007). Geriatric conditions and disability: the health and retirement study. *Annals of Internal Medicine*.

[32] Tinetti M. E., Inouye S. K., Gill T. M., Doucette J. T. (1995). Shared risk factors for falls, incontinence, and functional dependence: unifying the approach to geriatric syndromes. *The Journal of the American Medical Association*.

[33] Prados-Torres A., Calderón-Larrañaga A., Hancco-Saavedra J., Poblador-Plou B., van den Akker M. (2014). Multimorbidity patterns: a systematic review. *Journal of Clinical Epidemiology*.

[34] Violan C., Foguet-Boreu Q., Flores-Mateo G. (2014). Prevalence, determinants and patterns of multimorbidity in primary care: a systematic review of observational studies. *PLoS ONE*.

[35] Dong H. J., Wressle E., Marcusson J. (2013). Multimorbidity patterns of and use of health services by Swedish 85-year-olds: an exploratory study. *BMC Geriatrics*.

[36] Marengoni A., Rizzuto D., Wang H.-X., Winblad B., Fratiglioni L. (2009). Patterns of chronic multimorbidity in the elderly population. *Journal of the American Geriatrics Society*.

[37] Marengoni A., Bonometti F., Nobili A. (2010). In-hospital death and adverse clinical events in elderly patients according to disease clustering: the REPOSI study. *Rejuvenation Research*.

[38] John R., Kerby D. S., Hennessy C. H. (2003). Patterns and impact of comorbidity and multimorbidity among community-resident American Indian elders. *Gerontologist*.

[39] Cornell J. E., Pugh J. A., Williams J. W. (2007). Multimorbidity clusters: clustering binary data from multimorbidity clusters: clustering binary data from a large administrative medical database. *Applied Multivariate Research*.

[40] Goldstein G., Luther J. E., Jacoby A. M., Haas G. L., Gordon A. J. (2008). A taxonomy of medical comorbidity for veterans who are homeless. *Journal of Health Care for the Poor and Underserved*.

[41] Formiga F., Ferrer A., Sanz H., Marengoni A., Alburquerque J., Pujol R. (2013). Octabaix study members. Patterns of comorbidity and multimorbidity in the oldest old: the Octabaix study. *European Journal of Internal Medicine*.

[42] Simpson C. F., Boyd C. M., Carlson M. C., Griswold M. E., Guralnik J. M., Fried L. P. (2004). Agreement between self-report of disease diagnoses and medical record validation in disabled older women: factors that modify agreement. *Journal of the American Geriatrics Society*.

[43] Kriegsman D. M. W., Penninx B. W. J. H., van Eijk J. T. M., Boeke A. J. P., Deeg D. J. H. (1996). Self-reports and general practitioner information on the presence of chronic diseases in community dwelling elderly. A study on the accuracy of patients' self-reports and on determinants of inaccuracy. *Journal of Clinical Epidemiology*.

[44] The Italian Longitudinal Study on Aging Working Group (1997). Prevalence of chronic diseases in older Italians: comparing self-reported and clinical diagnoses. *International Journal of Epidemiology*.

[45] Tinetti M. E., Fried T. (2004). The end of the disease era. *American Journal of Medicine*.

[46] Collerton J. (2012). *Health and Disease in the Very Old: Findings from the Newcastle 85+ Study Newcastle upon Tyne*.

[47] Boyd C. M., Darer J., Boult C., Fried L. P., Boult L., Wu A. W. (2005). Clinical practice guidelines and quality of care for older patients with multiple comorbid diseases: implications for pay for performance. *The Journal of the American Medical Association*.

[48] Hughes L. D., McMurdo M. E. T., Guthrie B. (2013). Guidelines for people not for diseases: the challenges of applying UK clinical guidelines to people with multimorbidity. *Age and Ageing*.

[49] Smith S. M., Soubhi H., Fortin M., Hudon C., O'Dowd T. (2012). Managing patients with multimorbidity: systematic review of interventions in primary care and community settings. *British Medical Journal*.

[50] Van Spall H. G. C., Toren A., Kiss A., Fowler R. A. (2007). Eligibility criteria of randomized controlled trials published in high-impact general medical journals: a systematic sampling review. *The Journal of the American Medical Association*.

[51] Zulman D. M., Sussman J. B., Chen X., Cigolle C. T., Blaum C. S., Hayward R. A. (2011). Examining the evidence: a systematic review of the inclusion and analysis of older adults in randomized controlled trials. *Journal of General Internal Medicine*.

[52] Tinetti M. E., Bogardus S. T., Agostini J. V. (2004). Potential pitfalls of disease-specific guidelines for patients with multiple conditions. *The New England Journal of Medicine*.

[53] Cox L., Kloseck M., Crilly R., McWilliam C., Diachun L. (2011). Underrepresentation of individuals 80 years of age and older in chronic disease clinical practice guidelines. *Canadian Family Physician*.

[54] Haggerty J. L. (2012). Ordering the chaos for patients with multimorbidity. *British Medical Journal*.

[55] Kadam U. (2012). Redesigning the general practice consultation to improve care for patients with multimorbidity. *British Medical Journal*.

[56] Guthrie B., Payne K., Alderson P., McMurdo M. E. T., Mercer S. W. (2012). Adapting clinical guidelines to take account of multimorbidity. *British Medical Journal*.

[57] Muth C., van den Akker M., Blom J. W. (2014). The Ariadne principles: how to handle multimorbidity in primary care consultations. *BMC Medicine*.

[58] Vilà A., Villegas E., Cruanyes J. (2015). Cost-effectiveness of a Barcelona home care program for individuals with multimorbidity. *Journal of the American Geriatrics Society*.

[59] NHS England (2014). *NHS Five Year Forward View*.

