# Supplementary material for: Deconstructing Complex Multimorbidity in the Very Old: Findings from the Newcastle 85+ Study
Source: Biomed Res Int. 2016 Jan 13;2016:8745670. doi: 10.1155/2016/8745670 (PMC4738702; doi:10.1155/2016/8745670)
Supplement: Supplementary file 1 — Supplementary Material contains an appendix to the paper that gives further details of the sample selection and additional results tables (Supplementary Tables 1, 2, and 3). [file 8745670.f1.pdf]

APPENDIX: Deconstructing complex multimorbidity in the very old: findings from the Newcastle 85+ Study

FURTHER DETAILS OF SAMPLE SELECTION

The sampling frame for the Newcastle 85+ Study comprised all people born in 1921 who were permanently registered with a participating general practice in Newcastle upon Tyne or North Tyneside Primary Care Trusts (North-East England). We approached all 64 general practices in these trusts to participate in the study, and 82.8% (53/64) agreed. Practices who participated were similar to those who did not across key practice variables.[1] General practitioners were asked to review patient lists before mail-out and to exclude only those people with end stage terminal illness and those who might pose a safety risk to a nurse visiting alone. Excepting these exclusions, all those remaining in the sampling frame were sent a letter of invitation by the study team whether living at home or in an institution and regardless of their state of health. Recruitment and assessment took place over a 17 month period in 2006-7.

The recruitment profile is summarised in Supplementary Figure 1. Of the 1453 people eligible to participate in the Newcastle 85+ Study - that is registered with a participating general practice and still alive - 1042 (71.7%) were recruited. In total, 851 people (58.6% of those eligible) were recruited to health assessment plus review of general practice records, with an additional 188 (12.9%) recruited to record review only and 3 (0.2%) to health assessment only. The Newcastle 85+ cohort (health assessment sample) was socio-demographically representative of the local population, and of England and Wales, including the proportion in care homes.[1] The analysis reported required data from both the health assessment and general practice records. Exclusion of participants due to study withdrawal with request to destroy data (n=2) and incomplete general practice records (n=4) left 845 participants with this data, 58.2% of those eligible to participate. Complete data on all 15 diseases and 5 geriatric syndromes/impairments (termed geriatric conditions) was available for 710 of these participants (84.0%), who formed the sample for the principal analyses. Missing data arose from non-completion of questionnaires, electrocardiograms or blood tests. Comparison of the groups with and without complete data for all 20 conditions showed that those with missing data were more

likely to be female, to be resident in an institution, to have a higher prevalence of osteoporosis, urinary incontinence and cognitive impairment, and to be more disabled than those with complete data (Supplementary Table 1).

FURTHER INFORMATION ON MEASURES

The study questionnaires and the proforma used for the review of general practice records are available on the Newcastle 85+ Study website:

http://research.ncl.ac.uk/85plus/

REFERENCE

1. Collerton J, Davies K, Jagger C*, et al.* Health and disease in 85 year olds: baseline findings from the Newcastle 85+ cohort study. *BMJ* 2009; 339: b4904.

Supplementary Figure 1: Sample selection flow chart

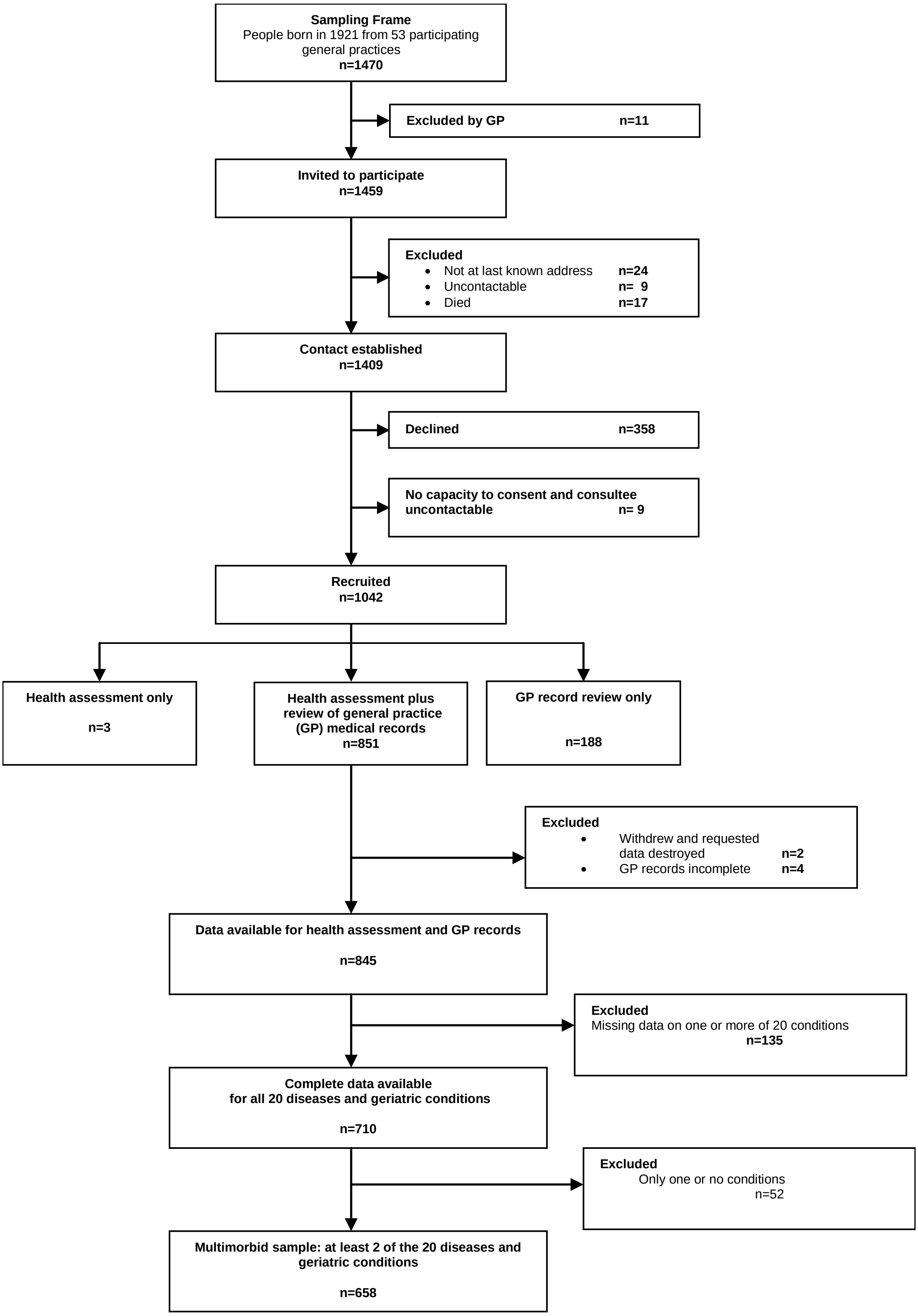

Supplementary Table 1: Characteristics of groups with and without complete data on all 20 conditions

	**Group with complete data on all 20 conditions N=710 (maximum*)**	**Group without complete data on all 20 conditions N=135 (maximum*)**	**P-value†**
Female, %(n)	59.9 (425)	74.8 (101)	**0.001**
Living in institution, %(n)	5.9 (42)	32.6 (44)	**<0.0001**
Years in full-time education, %(n)			0.653
0-9	64.7 (458)	62.8 (76)	
10-11	22.7 (161)	23.1 (28)	
12+	12.6 (89)	14.1 (17)	
Diseases, %(n)			
Hypertension	57.8 (410)	54.8 (74)	0.528
Ischaemic heart disease	36.1 (256)	42.3 (41)	0.234
Heart failure	11.1 (79)	15.6 (21)	0.144
Atrial fibrillation or flutter	13.5 (96)	21.5 (17)	0.054
Cerebrovascular disease	21.1 (150)	20.7 (28)	0.920
Peripheral vascular disease	7.3 (52)	5.2 (7)	0.371
Osteoarthritis	57.0 (405)	53.3 (72)	0.426
Inflammatory arthritis	3.8 (27)	6.7 (9)	0.131
Osteoporosis	12.1 (86)	19.3 (26)	**0.025**
Chronic obstructive pulmonary disease	16.5 (117)	17.0 (23)	0.837
Asthma	4.1 (29)	6.7 (9)	0.185
Diabetes mellitus	13.5 (96)	11.9 (16)	0.600
Thyroid disease	14.8 (105)	11.9 (16)	0.372
Cancer within 5 years	6.2 (44)	7.5 (10)	0.583
Renal impairment	23.8 (169)	24.6 (16)	0.883
Geriatric conditions, %(n)			
Urinary incontinence	31.3 (222)	42.4 (42)	**0.027**
Falls	17.2 (122)	21.8 (22)	0.258
Visual impairment	36.2 (257)	42.5 (48)	0.199
Hearing impairment	60.4 (429)	55.4 (72)	0.282
Cognitive impairment	6.9 (49)	21.5 (20)	**<0.0001**
Disability score‡, median (IQR)	3 (1 - 6)	8 (3 - 13)	**<0.0001**

*Sample size for groups with and without complete data on all 20 conditions may be less than the maximum specified due to missing data for comparator variables

†P-value for no difference between groups with/without complete data on all 20 conditions

‡Total number of activities of daily living performed with difficulty or requiring an aid/appliance or personal help[1]

Supplementary Table 2: Prevalence of diseases and geriatric conditions in complete case sample (N=710), by sex

	Men<br>% (n)	Women<br>% (n)	P-value*
**Diseases**			
Hypertension	53.3 (152)	60.7 (258)	0.051
Ischaemic heart disease	39.7 (113)	33.7 (143)	0.103
Heart failure	11.6 (33)	10.8 (46)	0.754
Atrial fibrillation or flutter	17.2 (49)	11.1 (47)	**0.019**
Cerebrovascular disease	24.2 (69)	19.1 (81)	0.099
Peripheral vascular disease	9.5 (27)	5.9 (25)	0.072
Osteoarthritis	47.4 (135)	63.5 (270)	**<0.0001**
Inflammatory arthritis	2.5 (7)	4.7 (20)	0.124
Osteoporosis	3.2 (9)	18.1 (77)	**<0.0001**
Chronic obstructive pulmonary disease	17.5 (50)	15.8 (67)	0.531
Asthma	2.5 (7)	5.2 (22)	0.073
Diabetes mellitus	15.4 (44)	12.2 (52)	0.221
Thyroid disease	6.3 (18)	20.5 (87)	**<0.0001**
Cancer within 5 years	8.1 (23)	4.9 (21)	0.090
Renal impairment	22.5 (64)	24.7 (105)	0.490
**Geriatric conditions**			
Urinary incontinence	22.5 (64)	37.2 (158)	**<0.0001**
Falls	17.9 (51)	16.7 (71)	0.681
Visual impairment	31.9 (91)	39.1 (166)	0.053
Hearing impairment	65.3 (186)	57.2 (243)	**0.031**
Cognitive impairment	6.3 (18)	7.3 (31)	0.614

*P-value for no difference between men and women

Supplementary Table 3: 20 diseases and geriatric conditions - proportion of cases with comorbidity and number of co-occurring conditions. Data are presented including and excluding geriatric conditions in the definition of co-occurring condition

	Total number of cases with index condition	Including geriatric conditions				Excluding geriatric conditions			
		Cases with comorbidity		Number of co-occurring conditions*		Cases with comorbidity		Number of co-occurring conditions*	
	N	N	% of all cases	Median	Inter-quartile range	N	% of all cases	Median	Inter-quartile range
**Diseases**									
Hypertension	410	396	96.6	4	3-5	384	93.7	2	2-4
Ischaemic heart disease	256	254	99.2	4	3-6	247	96.5	3	2-4
Heart failure	79	79	100.0	6	4-7	79	100.0	4	3-5
Atrial fibrillation or flutter	96	94	97.9	5	4-6	90	93.8	4	2-4
Cerebrovascular disease	150	148	98.7	5	3-6	144	96.0	3	2-4
Peripheral vascular disease	52	52	100.0	5	4-6	51	98.1	4	2-5
Osteoarthritis	405	397	98.0	4	3-5	367	90.6	3	2-4
Inflammatory arthritis	27	27	100.0	5	4-6	27	100.0	3	2-4
Osteoporosis	86	85	98.8	5	3-6	84	97.7	3	2-4
Chronic obstructive pulmonary disease	117	115	98.3	5	3-6	107	91.5	3	2-5
Asthma	29	28	96.6	4	3-6	28	96.6	3	2-4
Diabetes mellitus	96	96	100.0	5	3-6.5	94	97.9	3	2-5
Thyroid disease	105	104	99.1	5	3-6	101	96.2	3	2-4
Cancer within 5 years	44	44	100.0	5	3.5-7	43	97.7	4	3-5
Renal Impairment	169	169	100.0	5	4-6	166	98.2	4	2-4
**Geriatric conditions**									
Urinary incontinence	222	222	100.0	5	3-6	194	87.4	2	1-4
Falls	122	121	99.2	5	3-6	101	82.8	2	1-4
Visual impairment	257	257	100.0	4	3-5	216	84.1	2	1-3
Hearing impairment	429	417	97.2	4	3-5	354	82.5	2	1-4
Cognitive impairment	49	49	100.0	5	3-6	36	73.5	2	2-4

*The median number of co-occurring conditions is reported for those cases with comorbidity i.e. at least one condition in addition to the index disease
